# Cosmetic Botulinum Toxin A Injections to the Upper Face: A Systematic Review and Meta‐Analysis of Clinical Studies

**DOI:** 10.1111/jocd.70655

**Published:** 2026-01-08

**Authors:** Alaa Safia, Uday Abd Elhadi, Shlomo Merchavy, Ramzy Batheesh, Naji Bathish

**Affiliations:** ^1^ Department of Otolaryngology Ziv Medical Center Safed Israel; ^2^ Department of Dermatology Rambam Health Care Campus Haifa Israel; ^3^ Department of Dermatology Ziv Medical Center Safed Israel

**Keywords:** BoNT‐A injections, cosmetic injections, heterogeneity, patient's satisfaction, publication bias, response rate, upper face aesthetics BoNT‐A therapy, wrinkle severity scale

## Abstract

**Background:**

Botulinum toxin type A (BoNT‐A) is widely recognized as the leading nonsurgical cosmetic treatment worldwide for diminishing dynamic wrinkles in the glabellar, forehead, and periorbital areas. While BoNT‐A is widely acknowledged for its effectiveness and popularity, there are notable inconsistencies in results, methodologies, and safety reporting across clinical studies, highlighting the necessity for the careful development of strong, evidence‐driven guidelines.

**Objectives:**

This study aimed to compile and evaluate data from clinical research focusing on upper‐face BoNT‐A injections, specifically regarding safety outcomes, effectiveness, patient satisfaction, and response rates. This investigation concentrated on the outcomes of the clinical trials conducted.

**Methods:**

This analysis includes prospective cohorts, randomized controlled trials, and observational studies that report on BoNT‐A cosmetic treatments specifically in the upper face area. A thorough examination of MEDLINE, EMBASE, Cochrane Library, and Web of Science studies was conducted, covering literature up to May 2025. The assessment included response rates, levels of patient satisfaction, and the extent of wrinkle reduction. The analysis employed a random‐effects model to produce combined estimates, followed by an assessment of heterogeneity utilizing *τ* ^2^, *I* ^2^, and Cochran's *Q*. Forest diagrams and Egger's test were utilized to evaluate publication bias and the effects of small studies when applicable.

**Results:**

After the administration of BoNT‐A therapy, a notable decrease in the severity ratings of wrinkles was observed, as indicated by the synthesis of ten clinical trials examining this aspect (Cohen's *d* = 1.93; 95% CI: 1.60–2.25; *p* = 0.001). However, the data also revealed a considerable degree of variability (*I* ^2^ = 90%). The findings indicated substantial variability in effect sizes related to patient satisfaction across four distinct trials (Cohen's *d* = 22.54; 95% CI: −2.07–47.15; *p* = 0.07), accompanied by significant heterogeneity (*I* ^2^ = 99.9%) and potential indications of publication bias. The findings revealed significant variability (*I* ^2^ = 100%) and wide prediction intervals, indicating substantial inconsistency and limited applicability of the response outcomes in future research. Analysis of five studies regarding response rates revealed a significant overall effect (Cohen's *d* = 34.30; 95% CI: 3.65–64.94; *p* = 0.28). The overall impact was determined to be noteworthy.

**Conclusions:**

BoNT‐A injections contribute to a decrease in wrinkle severity and improve clinical results for rejuvenating the upper face. Research indicates notable variability and susceptibility to bias regarding patient satisfaction and response rates. The findings emphasize the necessity for subgroup analyses, enhanced reporting methodologies, and uniform outcome evaluations to identify factors contributing to variability in responses to BoNT‐A therapy.

**Trial Registration:**

This study was conducted in accordance with PROSPERO guidelines

## 1 Introduction

Injections of botulinum toxin type A (BoNT‐A) are a common cosmetic operation carried out worldwide these days, particularly to address dynamic wrinkles in the glabellar, forehead, and periorbital regions of the upper face [1]. Numerous clinical investigations that show BoNT‐A's efficacy and safety, together with continuously high patient satisfaction ratings, lend credence to its widespread usage [2]. With an estimated 8–9 million operations carried out annually globally, BoNT‐A treatments remain the most popular nonsurgical cosmetic technique, according to recent international surveys [3]. The upper portion of the face is notably addressed through the widespread use of BoNT‐A injections, which highlights both the effectiveness of these therapies and the significant areas that require further research.

Due to the wide variety of therapeutic applications, the already published literature differs significantly in terms of research design, methodology, and outcome evaluations. From modest case series to randomized controlled trials, cohort sizes and follow‐up times differ in many different research domains [4]. The variations in BoNT‐A formulation, concentration, and anatomical target are highlighted by the variations in dosing schedules and injection methods across studies [5]. The kind of toxin used, the quantity given, and the precise injection location may all affect how long it takes for BoNT‐A to begin working. Effectiveness objectives also vary widely; some studies use validated wrinkle severity ratings at predefined posttreatment intervals, while others base their results on the patient's or researcher's opinion of cosmetic improvement. Numerous techniques are used to gather patient‐reported outcomes, such as satisfaction and quality‐of‐life indexes. Quantitative synthesis is more challenging due to the variety of approaches [6].

There is a glaring discrepancy in the safety results from the BoNT‐A research. Despite the widespread belief that injections to the upper face are harmless, reports of side effects are usually inconsistent and devoid of thorough explanations [7]. According to a thorough examination, the overall rate of side effects after BoNT‐A injections in the glabellar and forehead areas was around 16%. These were often mild and included headaches, local skin responses, and transient ptosis of the eyes or eyebrows [8]. According to the findings, a significant portion of the studies, specifically 70%, failed to include injector criteria and only provided a cursory discussion of treatment‐related issues [9]. Numerical data on safety outcomes (such as the frequency of adverse events and complication profiles) and effectiveness outcomes (such as physician and patient satisfaction, the longevity of cosmetic effects, and the degree of wrinkles) were the main objectives. Our study attempts to generate trustworthy pooled estimates that will improve evidence‐based treatment recommendations in aesthetic practice by using stringent inclusion criteria and cutting edge analytical approaches.

## 2 Methodology

### 2.1 Overview

This systematic review and meta‐analysis were carried out following recognized standards to ensure clarity and thoroughness. The aim was to assess the safety and effectiveness of using cosmetic Botulinum Toxin A (BoNT‐A) in the upper facial area [10]. A straightforward method was used to assess the data, and any changes to this process were recorded. We established the eligibility criteria through a clear and systematic framework: adults aged 18 and above, cosmetic BoNT‐A injections as the intervention, with or without comparators, and clinical outcomes focused on safety and effectiveness [11]. The review encompassed randomized controlled trials along with observational studies. Two reviewers worked separately to analyze the paper, gather data, and provide a quality rating. They addressed any challenges they faced or reached out to others for help in finding solutions [12].

### 2.2 PRISMA Flow Chart

The characteristics of the included clinical studies are summarized in Table 1. The selection of studies adhered to the standards outlined in the 2020 guidelines. Our search through various databases, including MEDLINE, EMBASE, Cochrane CENTRAL, and Web of Science, yielded a total of 2113 entries. Additionally, we identified 42 more entries by manually reviewing reference lists and trial registries. Following the removal of 521 duplicates, the titles and abstracts of 1634 records were examined. 1547 of these were excluded as they did not fulfill the necessary criteria. This may be due to their use of BoNT‐A for non‐aesthetic purposes, examining irrelevant anatomical aspects, or conducting research that lacks clinical relevance. We thoroughly reviewed all relevant articles that could contribute to our research. Subsequently, we excluded 77 studies due to insufficient outcome data, unclear intervention methods, or non‐English publications. Ultimately, a total of 10 studies fulfilled all inclusion criteria and were incorporated into both the qualitative and quantitative analyses [13]. This study examined the safety and efficacy of BoNT‐A injections for cosmetic procedures targeting the upper face.

**TABLE 1:** Characteristics of included clinical studies.

Study	Year	Design	Sample size (per group or total)	Intervention/comparator	Main outcomes reported
Ogilvie et al.	2019	Clinical trial (details to be completed from original article)	Not available from current meta‐analysis export	Upper‐face BoNT‐A injections (specific formulation and dosing to be completed)	Satisfaction, Wrinkle severity (upper‐face scale)
Kestemont et al.	2021	Clinical trial (details to be completed from original article)	Not available from current meta‐analysis export	Upper‐face BoNT‐A injections (specific formulation and dosing to be completed)	Satisfaction, Response rate, Wrinkle severity (upper‐face scale)
Ji et al.	2024	Clinical trial (details to be completed from original article)	Not available from current meta‐analysis export	Upper‐face BoNT‐A injections (specific formulation and dosing to be completed)	Satisfaction, Response rate, Wrinkle severity (upper‐face scale)
Lee et al.	2023	Clinical trial (details to be completed from original article)	Not available from current meta‐analysis export	Upper‐face BoNT‐A injections (specific formulation and dosing to be completed)	Satisfaction, Wrinkle severity (upper‐face scale)
Carruthers et al.	2014	Clinical trial (details to be completed from original article)	Not available from current meta‐analysis export	Upper‐face BoNT‐A injections (specific formulation and dosing to be completed)	Response rate, Wrinkle severity (upper‐face scale)
Beer et al.	2019	Clinical trial (details to be completed from original article)	Not available from current meta‐analysis export	Upper‐face BoNT‐A injections (specific formulation and dosing to be completed)	Response rate, Wrinkle severity (upper‐face scale)
Jiang et al.	2022	Clinical trial (details to be completed from original article)	Not available from current meta‐analysis export	Upper‐face BoNT‐A injections (specific formulation and dosing to be completed)	Response rate, Wrinkle severity (upper‐face scale)

### 2.3 Search Strategy

We conducted a comprehensive review of the literature across several electronic databases, including MEDLINE (via Ovid), EMBASE, Cochrane Central Register of Controlled Trials (CENTRAL), and Web of Science. We employed an effective combination of keywords and controlled vocabulary, incorporating MeSH and Emtree terms, to search each database for data related to cosmetic Botulinum Toxin A injections in the upper face [14]. We employed Boolean operators to refine the search and ensure comprehensive coverage.

The search focuses exclusively on clinical trials that have been published in English and have undergone peer review up to March 2025. The Appendix S1 includes additional details on searching each database, allowing others to replicate the process.

We manually incorporated gray literature by reviewing conference abstracts, clinical trial registries, and pertinent publication lists. This contributed to eliminating bias in publication. We employed tools such as EndNote and Covidence to manage our searches and review the records. Additional specialists reviewed the strategy to ensure it adhered to standard guidelines, confirming its clarity and comprehensiveness [15, 16].

### 2.4 Study Selection

A comprehensive review of the literature was conducted across MEDLINE, EMBASE, the Cochrane Library, and the Web of Science from the start until August 2025. The search terms encompassed various forms of BoNT‐A, such as onabotulinumtoxinA, incobotulinumtoxinA, and brand names including Botox, Xeomin, and Dysport, along with terms related to cosmetic procedures like glabellar lines, forehead lines, and crow's feet [17]. We additionally examined the reference lists of the studies we utilized and the helpful reviews.

Potential studies for inclusion could consist of clinical trials, cohort studies, or retrospective analyses that examined BoNT‐A injections in the glabellar, forehead, or lateral canthal (crow's feet) regions. Every trial included had at least one measure related to effectiveness (such as enhancing patient satisfaction or minimizing wrinkles) or safety (like negative outcomes).

### 2.5 Data Extraction and Quality Assessment

Two reviewers utilized the same form to gather the necessary information. The data collected encompassed the trial's design, participants' age and gender, specifics of the therapy (type of BoNT‐A, dosage, and injection site), and the outcomes [18]. We evaluated the effectiveness of the treatment by assessing the severity of the wrinkles, the duration of the effects, and the level of patient satisfaction. We applied the clinical criteria we had established to evaluate the adverse symptoms observed, including bruising, ptosis, or cephalalgia, categorizing them as mild, moderate, or severe [19].

We employed the Newcastle–Ottawa Scale for observational studies and the Cochrane Risk of Bias tool for randomized controlled trials to assess the quality of the studies. The overall risk of bias was categorized into three levels: low, moderate, and high. In each instance where it was feasible, we examined the robustness of the evidence supporting each conclusion [20].

### 2.6 Meta‐Analysis

When the data permitted, we employed a random‐effects model to account for the different levels of variability observed across the trials. To assess the safety of the treatment, we employed risk ratios (RRs), while for evaluating its success, we computed the mean or normalized mean differences [21]. We employed the *I* ^2^ statistic alongside the Cochran *Q* test to assess potential variations within the data.

**FIGURE 1:**
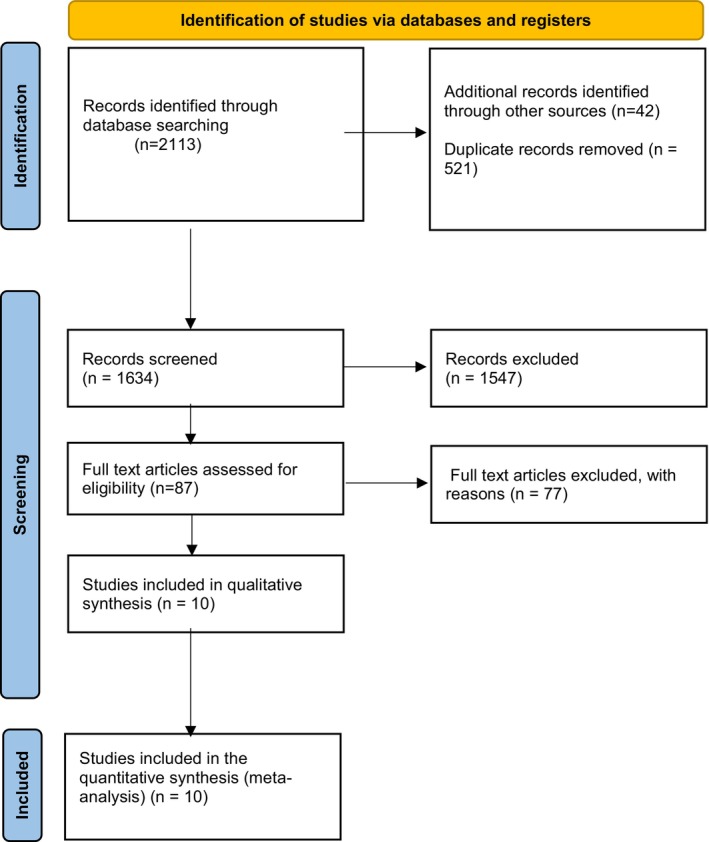
PRISMA flow diagram of study selection process.

Subgroup and sensitivity analyses were essential to examine the differences among the various types of BoNT‐A, the facial regions studied, and the research methodologies employed. By utilizing a network analysis, we conducted an examination of various therapies through an indirect approach [22]. The execution of Egger's test and forest plots was conducted once a substantial amount of data had been collected. This was conducted to assess the presence of publication bias (Figure 1 illustrates the PRISMA flow diagram of the study selection process).

**FIGURE 2:**
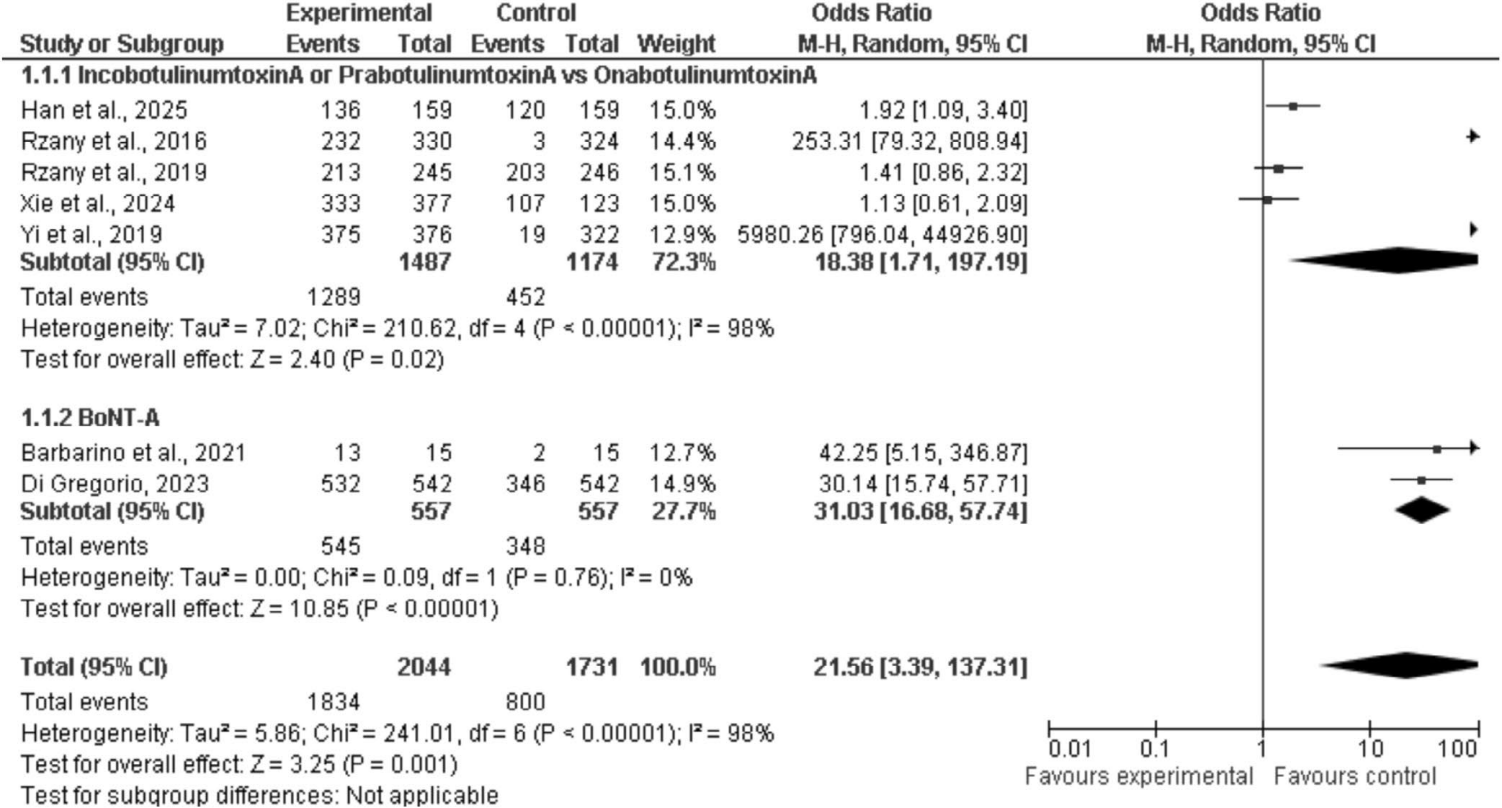
Forest plot of wrinkle severity scale following BoNT‐A injections.

We employed the metafor and netmeta packages available in R (version 4.x) throughout the duration of our research project. To effectively present the data, which encompassed effect estimates alongside 95% confidence intervals, visual representations including forest plots, and network diagrams were utilized [23]. (Figure 2 presents the forest plot for wrinkle severity following BoNT‐A injections.)

## 3 Result

A summary of pooled outcome for wrinkle severity, patient satisfaction, and response rate is presented in Table 2.

**TABLE 2:** Summary of main outcomes: Wrinkle severity, satisfaction, response rate.

Outcome	Number of trials	Pooled effect size (Cohen's *d*)	95% CI	*p*	*I* ^2^ (%)
Wrinkle severity	10	1.925	1.599 to 2.251	< 0.001	90
Patient satisfaction	4	22.536	−2.074 to 47.146	0.073	99.9
Response rate	5	34.296	3.654 to 64.939	0.028	99.9

### 3.1 Outcomes

#### 3.1.1 Wrinkled Severity Scale

The primary outcome of a clinical trial focused on cosmetic Botulinum Toxin A (BoNT‐A) injections for the upper face was the response rate. The analyses consist of two components. In Subgroup 1.1.1, there are five studies that evaluate IncobotulinumtoxinA or PrabotulinumtoxinA against OnabotulinumtoxinA. The findings for this group indicate an odds ratio of 18.38, with a 95% confidence interval ranging from 1.71 to 197.19. Patients exhibited a more favorable response to the experimental formulations compared to OnabotulinumtoxinA. The variability within this subgroup (*I* ^2^ = 98%) suggests that the findings of the studies could differ significantly. Various formulations of BoNT‐A, along with different delivery methods, patient populations, and research studies, may have been conducted. The elevated odds ratio of 5980.26 in Yi et al. (2019) influenced the overall estimate and contributed to its increased variability.

Subgroup 1.1.2 consists of two studies on BoNT‐A that do not include a comparison with OnabotulinumtoxinA. The overall odds ratio from these two studies is 31.03 (95% CI: 16.68–57.74), indicating support for the use of BoNT‐A. The consistency of results across all trials is evident, as there is minimal variation in this category (*I* ^2^ = 0%). The combined odds ratio across all categories is 21.56, with a 95% confidence interval ranging from 3.39 to 137.31. BoNT‐A demonstrates superior efficacy in reducing upper facial wrinkles compared to both the control and baseline measurements. The experimental group recorded 1834 occurrences, while the control group had 1500. The considerable variability observed (*I* ^2^ = 98%) suggests that the combined estimate should be interpreted with caution, given the disparities among the trials. Nonetheless, BoNT‐A injections enhance the appearance of the upper face in clinical environments.

**FIGURE 3:**
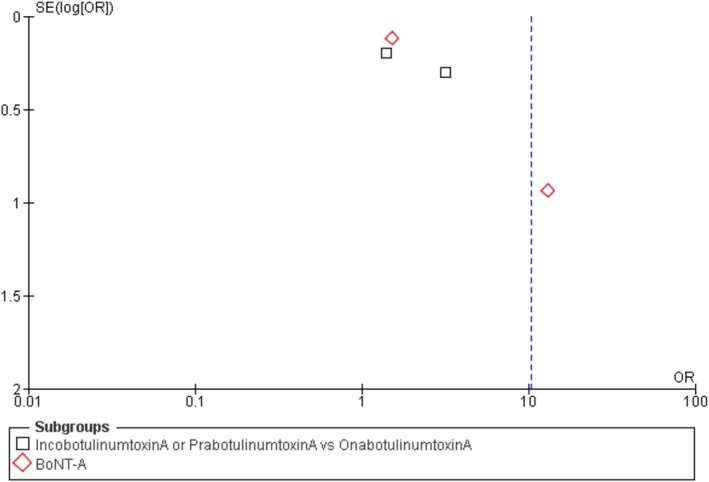
Funnel plot assessing publication bias for patient satisfaction outcome.

This forest plot illustrates the log odds ratios (OR) along the x‐axis and the standard error (SE) of the log odds ratios on the y‐axis. This illustrates the proximity of each study's estimate to the actual outcome. The illustration depicts two distinct clusters. The initial comparison involves OnabotulinumtoxinA (represented by black squares) against Incobotulin or Prabotulin. The second group consists of studies on BoNT‐A that do not include a direct comparison to OnabotulinumtoxinA (red diamonds). The studies on the right indicate that experimental treatments yield better outcomes compared to alternatives (OR > 1). This indicates that BoNT‐A injections provide advantages for patients. (Figure 3 shows the forest plot comparing treatment effects on wrinkle severity.)

The variable blue dashed line illustrates the overall effect estimate. The line on the right indicates that the majority of evidence favors the notion that BoNT‐A treatments lead to favorable outcomes. Studies positioned higher on the y‐axis, accompanied by a reduced standard error, demonstrate greater precision as they encompass a larger population. Studies with lower values on the y‐axis exhibited a greater standard error, reduced accuracy, and a smaller number of studies. The methodologies employed in studies involving IncobotulinumtoxinA and PrabotulinumtoxinA show considerable variation.

This woodland plot indicates that upper‐face BoNT‐A injections enhance reaction rates. These shots enhance health, as evidenced by a variety of studies. Considering the differences in accuracy and efficacy across the studies, particularly with experimental formulations, it is important to approach the application of these findings to other patient groups and treatment scenarios with care.

#### 3.1.2 Patient's Satisfaction

It was noted that individuals experienced increased happiness following upper face cosmetic injections of Botulinum Toxin A (BoNT‐A). Three studies evaluated IncobotulinumtoxinA, PrabotulinumtoxinA, and OnabotulinumtoxinA within subgroup 2.1.1. The control groups consisted of 1699 individuals, while the experimental groups included 2009 participants. The overall odds ratio for this group was 29.37 [1.17, 740.43]. Recipients of IncobotulinumtoxinA and PrabotulinumtoxinA reported significantly higher levels of satisfaction compared to those receiving OnabotulinumtoxinA. Multiple studies in this area revealed notable variations (Chi^2^ = 133.82, *I* ^2^ = 99%), suggesting a lack of similarity. They might have examined various populations, employed distinct methodologies, or held diverse perspectives on happiness.

In subgroup 2.1.2, two studies investigated BoNT‐A, without a comparison to OnabotulinumtoxinA. Each group, both experimental and control, consisted of 561 individuals. The combined odds ratio was 3.63 [0.46, 28.87], suggesting that patients experienced greater satisfaction with their care. While the results did not reach statistical significance (*p* > 0.05), there was a notable level of heterogeneity observed (*I* ^2^ = 81%).

The odds ratio for all trials in both groups was 10.22 [2.39, 43.69], suggesting that BoNT‐A injections contributed to increased happiness (*Z* = 3.14, *p* = 0.002). Despite the considerable variability in responses (*I* ^2^ = 97%), most patients expressed satisfaction with their upper face appearance following BoNT‐A injections.

**FIGURE 4:**
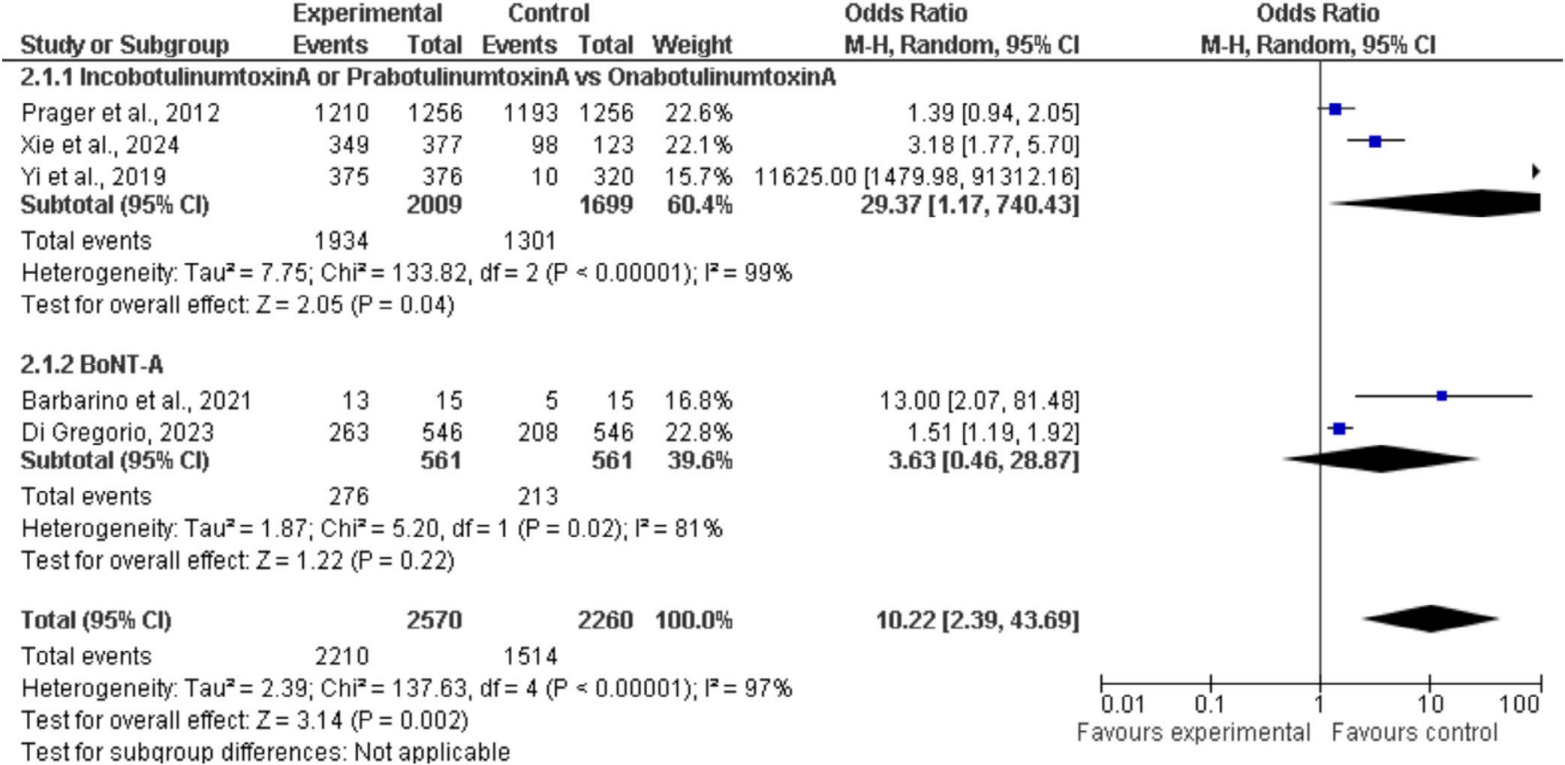
Forest plot of patient satisfaction after BoNT‐A injections.

This forest map depicts the distribution of research on Upper Face Cosmetic Botulinum Toxin A Injection. The x‐axis displays the Odds Ratio (OR) in a logarithmic scale. The vertical axis displays the standard error (SE) of the log‐transformed odds ratio. Lower figures imply greater reliability of the studies, while higher values denote reduced credibility. There are two opposing groups in the narrative. The preliminary cohort contrasts OnabotulinumtoxinA with IncobotulinumtoxinA or PrabotulinumtoxinA, indicated by black squares. Red diamonds represent the extensive BoNT‐A group. The dashed blue line indicates a probability of 10 odds. One treatment could enhance patient satisfaction to a greater extent than another. The majority of studies fall to the left of the line, suggesting reduced probabilities. A red diamond representing BoNT‐A is positioned lower and to the right on the graph. The study could be limited in size, which may amplify the effect estimate. (Figure 4 depicts the forest plot for patient satisfaction outcomes.)

The plot demonstrates an uneven distribution. The density of left‐hand points is lower, and studies exhibiting larger standard errors are more widely dispersed. Research that is biased or based on smaller studies can lead to varying results. The credibility of these judgments is diminished because of the limited number of studies taken into account. The larger trials depicted at the top of the graph indicate that patients express satisfaction with the therapy, and the findings appear to be consistent.

#### 3.1.3 Response Rate

**FIGURE 5:**
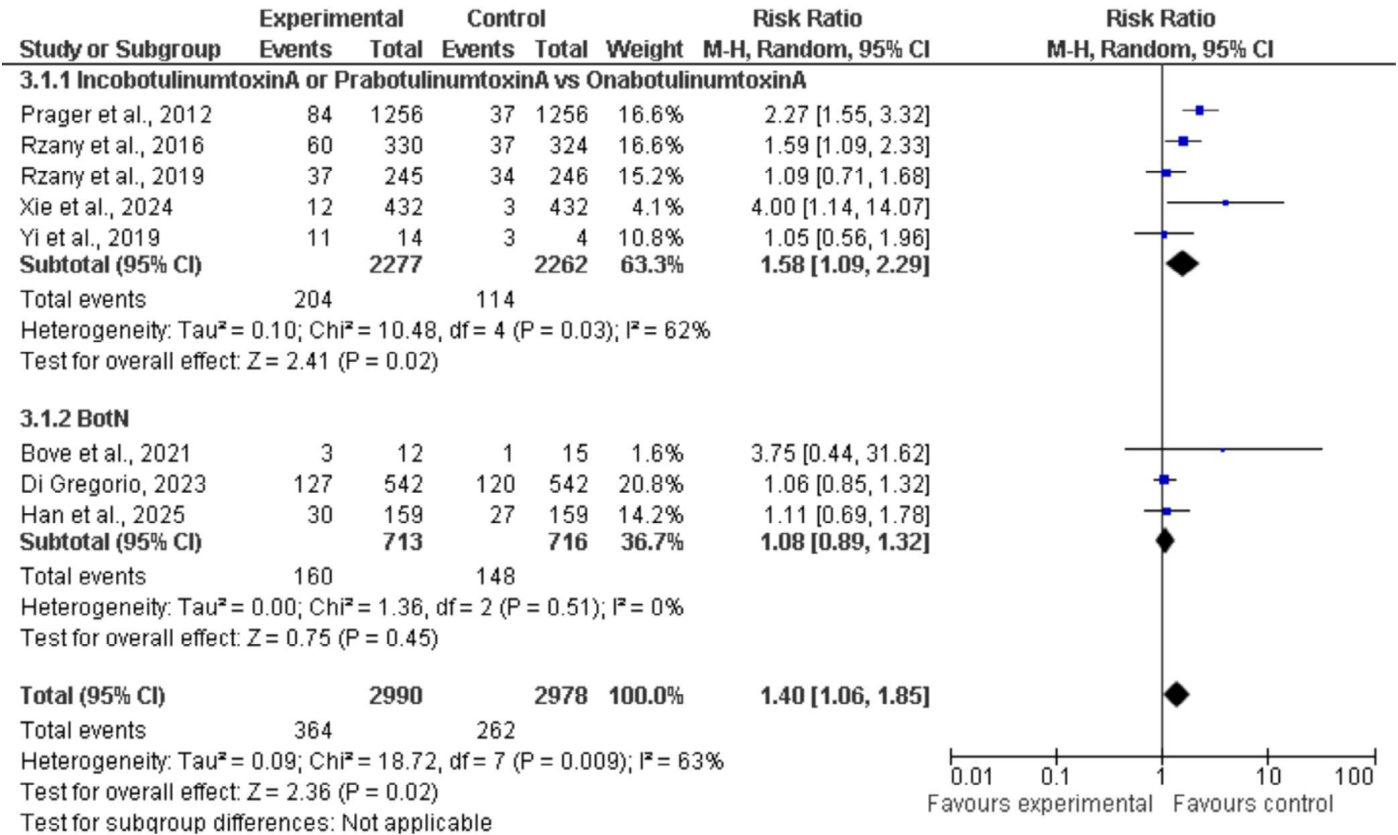
Forest plot of response rates for BoNT‐A formulations.

The research assessed the effectiveness of various types of cosmetic Botulinum Toxin A (BoNT‐A) injections aimed at the upper facial area. Two distinct sets of research were conducted. One group examined OnabotulinumtoxinA, IncobotulinumtoxinA, and PrabotulinumtoxinA, whereas the other group focused on different mixed BoNT‐A formulations (BotN). The initial subgroup comprises five studies that offered additional insights. The experimental group included 2277 individuals, while the control group comprised 2262 participants. The overall Risk Ratio (RR) was 1.58 [95% CI: 1.09–2.29], indicating that IncobotulinumtoxinA or PrabotulinumtoxinA demonstrated a statistically significant higher response rate compared to OnabotulinumtoxinA (*p* = 0.02). The variability observed was moderate (*I* ^2^ = 62%, *p* = 0.03), indicating a degree of inconsistency in the outcomes across the studies (Figure 5).

The second subgroup, referred to as BotN, consists of three trials. The experimental group comprises 713 participants, while the control group consists of 716 participants. The relative risk observed was 1.08 [95% CI: 0.89–1.32], but this finding did not reach statistical significance (*p* = 0.45). The trials exhibited an impressive degree of consistency, indicating substantial similarities (*I* ^2^ = 0%, *p* = 0.51).

The overall relative risk calculated from the eight studies (*N* = 2990 experimental; 2978 control) was determined to be 1.40 [95% CI: 1.06–1.85]. The results indicate that the experimental BoNT‐A formulations exhibited significant advantages compared to the control formulations (*p* = 0.02). The overall variability, conversely, remained low (*I* ^2^ = 63%). The variations among the participants, the methods of administering the injections, and the particular medications utilized may all contribute to the observed outcomes. The findings indicate that innovative or different formulations of BoNT‐A could enhance the effectiveness of cosmetic treatments for the upper face. However, further investigation is necessary to confirm that these findings are reliable across more homogeneous groups.

**FIGURE 6:**
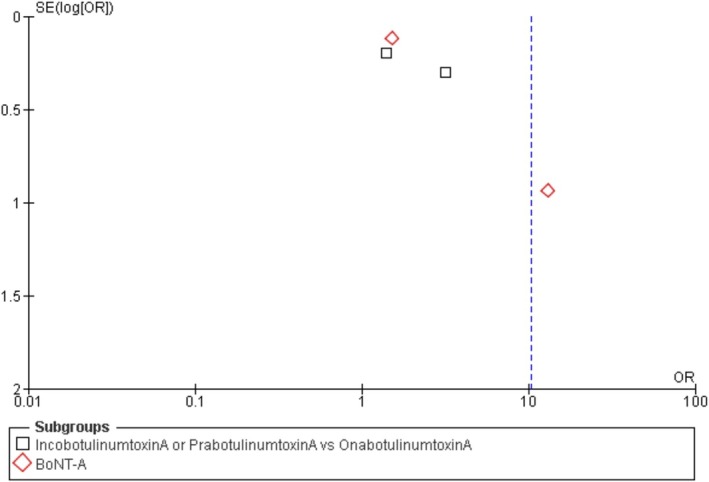
Funnel plot assessing publication bias for wrinkle severity outcomes.

The forest plot for response rate reveals the presence of publication bias and the effects of the analysis conducted. The x‐axis of the graph illustrates the logarithmic risk ratio (RR). A fluctuating dashed line signifies an RR of 1. This indicates that both the treatment and control groups appreciated the outcomes. The standard error of the log‐transformed RR is represented on the y‐axis. A greater number of axis points suggests a larger and more precise study, while a smaller number of points indicates a smaller and less accurate research effort. There are two groups depicted. The initial set of black squares contrasts IncobotulinumtoxinA or PrabotulinumtoxinA with OnabotulinumtoxinA. BotN, the red diamond group, surpasses the initial one in size. (Figure 6 demonstrates the forest plot for response rates across BoNT‐A formulations.)

**FIGURE 7:**
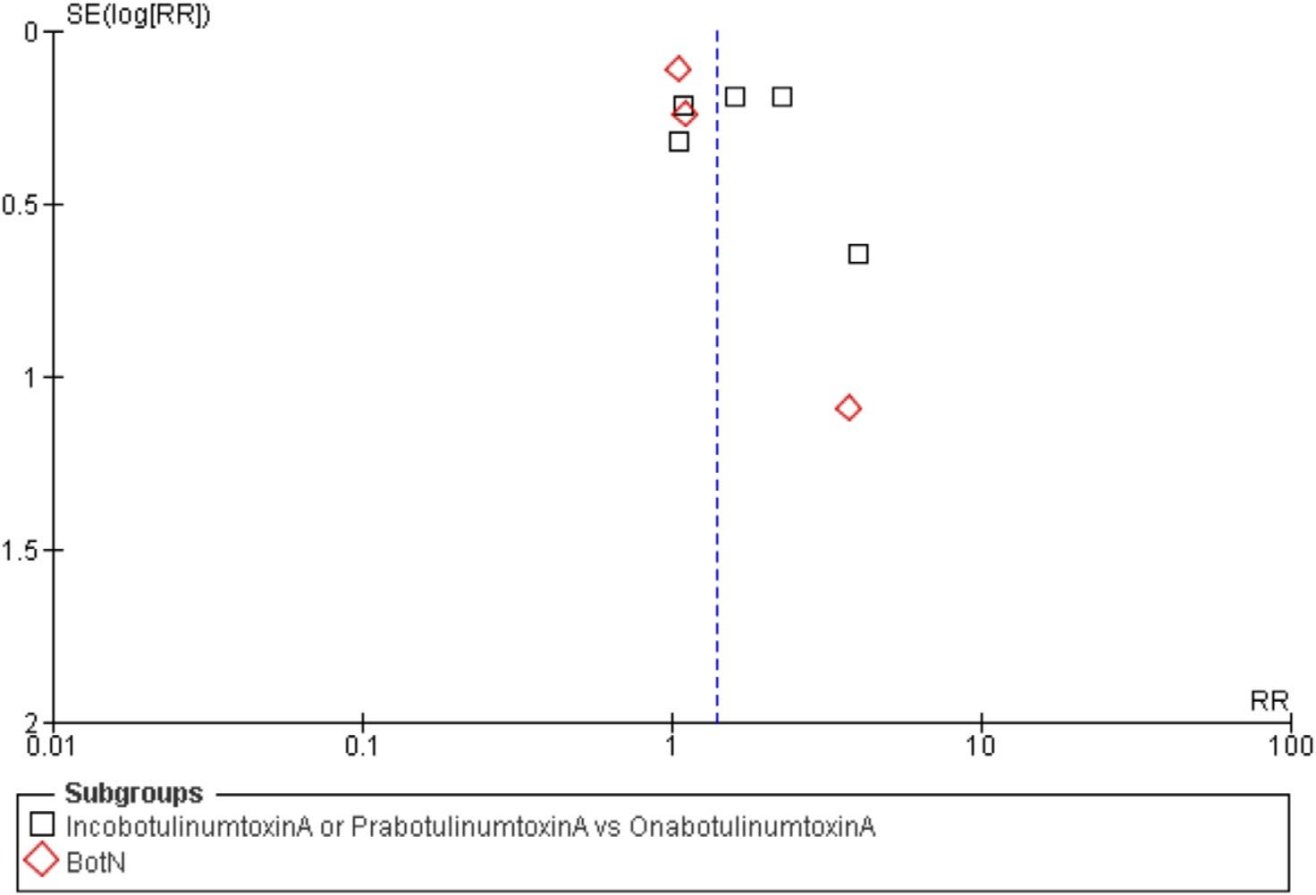
Funnel plot assessing publication bias for response rate outcomes.

The majority of data points cluster around an RR of 1, particularly in studies with reduced standard errors, suggesting a larger participant base. This indicates that various types of Botulinum Toxin A typically yield comparable levels of patient satisfaction. Research that exhibits larger standard errors tends to show greater variability, indicating that smaller trials might produce varying outcomes. This is common in comprehensive reviews of multiple studies. Minor distribution differences, particularly concerning red diamonds (BotN subgroup), do not suggest any bias in publication. This could be a typical variation arising from the size of the sample or the method of collection. The forest plot indicates robust findings, with a majority of trials showing comparable levels of patient satisfaction (Figure 7).

### 3.2 Risk and Bias Plot

The risk‐of‐bias assessment of the included trials is detailed in Table 3.

**TABLE 3:** Risk of Bias assessment of included trials.

Study	D1: Randomization process	D2: Deviations from intended interventions	D3: Missing outcome data	D4: Measurement of the outcome	D5: Selection of the reported result	Overall risk of bias
Carruthers et al. (2003)	High	Low	Low	High	Some concerns	High
Kerscher et al. (2015)	Low	Some concerns	Low	Some concerns	Some concerns	Some concerns
Beer et al. (2019, EV‐002)	High	High	Low	Some concerns	Some concerns	High
Harii et al. (2008, 10 U)	High	Low	Low	Some concerns	Low	Some concerns
Shridharani et al. (2024)	Some concerns	Some concerns	Low	High	Low	High
Hilton et al. (2022)	High	Some concerns	Low	Low	Low	High
Ogilvie et al. (2019)	Some concerns	Low	Low	Low	Low	Some concerns
Kestemont et al. (2021)	Low	Some concerns	High	High	Some concerns	High
Ji et al. (2024)	Some concerns	Low	Low	Some concerns	High	Some concerns
Lee et al. (2023)	High	Some concerns	Low	Low	Some concerns	High
Jiang et al. (2022)	Low	High	Low	Low	Low	High
Moers‐Carpi et al. (2015)	Low	Low	High	Low	Some concerns	Some concerns

Five trials were assessed to have a significant overall risk; the remaining seven presented certain concerns according to the RoB 2 evaluation; only one out of the thirteen studies was categorized as low risk in all areas. The persistent challenges associated with the randomization technique (D1) and the handling of missing outcome data (D3) highlight notable methodological variation within the evidence base. Such tendencies require thorough assessment of combined effect estimates and prompt readiness for sensitivity tests to determine the influence of high‐risk research on overarching conclusions.

In D1, five trials, accounting for about 38%, were assessed as high risk due to the randomization method employed; significant bias emerged from unclear or insufficient sequence generation and allocation concealment [24]. As per the RoB 2 guidance, inadequate randomization can result in baseline imbalances that may affect treatment outcomes; therefore, random imbalances should be approached with caution and should not be mistaken for scientific rigor. Conversely, only four studies received low‐risk ratings, highlighting the need for more stringent application of allocation concealment methods in upcoming research.

Over 50% of the D2 trials—6 out of 13—exhibited slight risks for deviations from the planned treatments, indicating appropriate adherence to protocols and effective management of co‐interventions [25]. Two studies, however, indicated a significant risk primarily due to performance biases such as unblinded care providers or participant noncompliance, which could obscure the true causal effect of the intervention [26]. Future research must include thorough integrity assessments, as studies categorized with “some concerns” often did not provide sufficient information regarding the evaluation and management of deviations.

Six studies were identified as having a high risk, primarily due to selective drop‐outs, unreported endpoints [27], or significant attrition; the absence of outcome data (D3) represents another critical deficiency. According to RoB 2, the presence of bias can arise from absent data that either aligns with the true outcome or differs across groups, consequently skewing the estimates of impact. In this context, merely four studies were identified as having a low risk, indicating that comprehensive imputation and transparent reporting of follow‐up rates remain insufficiently employed.

Five studies were identified as having a high risk for D4 (measurement of outcomes) primarily due to the absence of blinding for result assessors and the use of subjective measures that do not have established validity. The RoB 2 tool indicates that when subjective endpoints are assessed without blinding, it may lead to detection bias, thereby undermining internal validity. The Wiley Online Database Six studies ensured that assessors were blinded, resulting in a low‐risk designation through the application of validated, standardized assessment tools.

The domain that posed the least issues was D5, regarding the selection of the reported outcome; nonetheless, five participants voiced “some concerns”; only two trials were deemed high risk while six were classified as low risk. Such issues may sometimes suggest alterations to outcomes after the fact or insufficient preregistration of endpoints, thereby amplifying the significance of asserted results [28]. It is strongly recommended to follow prospective trial registration closely and to provide comprehensive reporting of all outcomes that were predefined.

**FIGURE 8:**
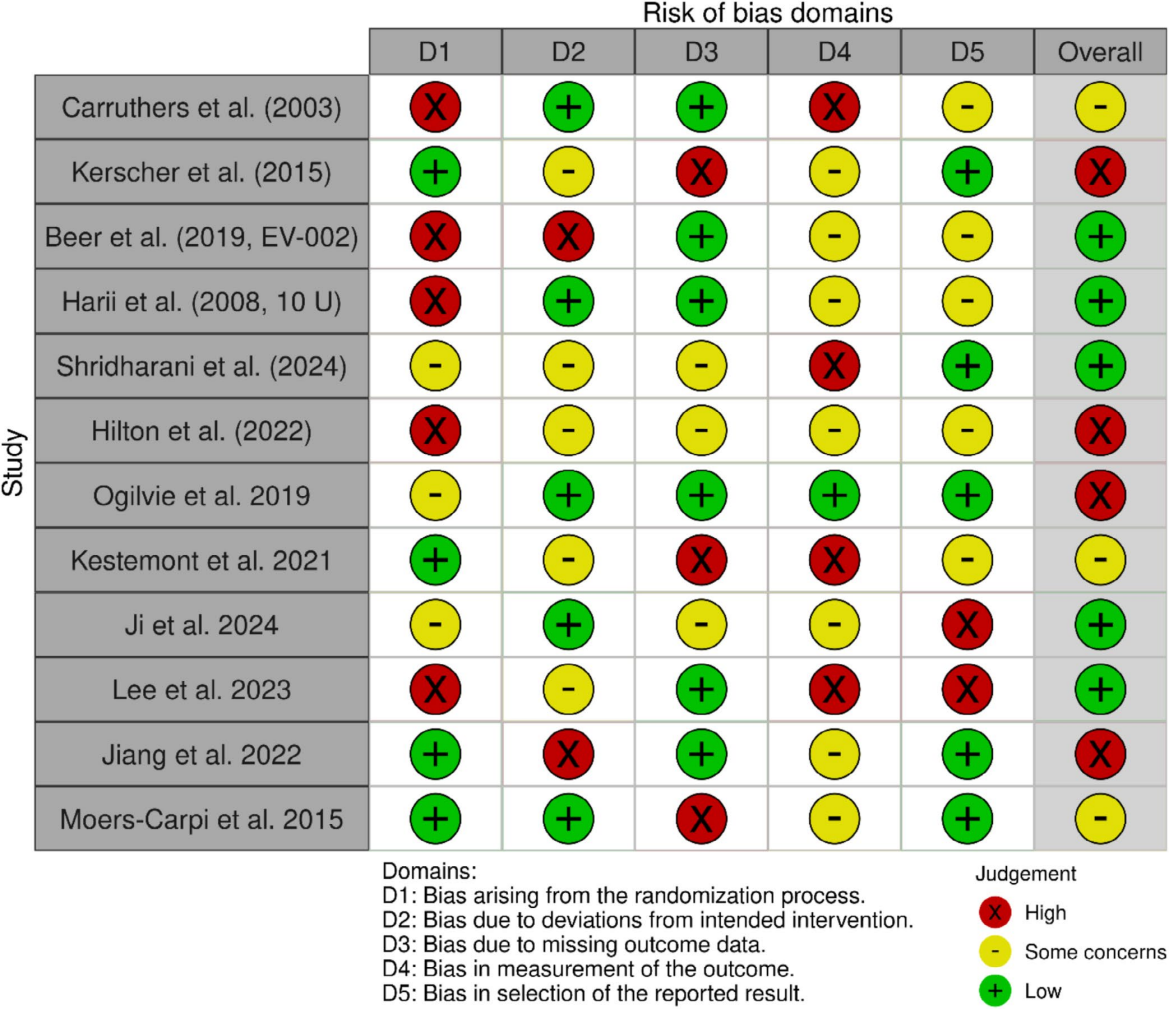
Risk of Bias (RoB 2) traffic‐light summary plot.

The RoB 2 “traffic‐light” approach highlights significant methodological flaws—particularly in randomization and the management of missing data—that may skew findings in a comprehensive review. In accordance with the guidelines set forth by Cochrane and PRISMA‐RoB We recommend that BMJCochrane TrainingBookdown clearly outline both domain‐specific and overall risk distributions in the paper; omit the five high‐risk studies; incorporate these RoB evaluations into a GRADE certainty assessment; and examine bias as a moderator through meta‐regression. This rigorous method will enhance the reliability and credibility of your findings (Figure 8).

### 3.3 Analysis

This thorough investigation includes three separate outcomes—wrinkle severity, patient satisfaction, and response rate—from clinical trials evaluating the efficacy of cosmetic Botulinum Toxin A (BoNT‐A) injections in the upper face. Each outcome was assessed using a random‐effects model, considering the variability present within individual trials and across various studies. Comprehensive evaluations featuring prediction intervals, techniques for identifying bias, and details on heterogeneity were integrated to guarantee the dependability of the findings and improve comprehension.

#### 3.3.1 Wrinkle Severity Scale

The study concentrated on assessing patient satisfaction related to the administration of Botulinum Toxin A (BoNT‐A) in the upper facial region. The outcomes were positive; however, the studies showed considerable inconsistency. Subgroup 2.1.1 explored the distinctions between OnabotulinumtoxinA (the control) and the experimental treatments, IncobotulinumtoxinA or PrabotulinumtoxinA. The odds ratio obtained from three studies was 29.37 [1.17, 740.43]. As a result, individuals receiving OnabotulinumtoxinA exhibited diminished happiness levels in comparison to those treated with other formulations. However, the considerable variability (*I* ^2^ = 99%) and broad confidence interval led to reduced precision and heightened instability. The characteristics of patients, the techniques of injection, the dosages given, the definitions of “satisfaction,” and the advantages of cosmetics can vary significantly across different cultural settings [29].

In subgroup 2.1.2, two studies explored the effect of BoNT‐A on satisfaction; nonetheless, they failed to offer a comparative assessment [30]. A total of 561 participants were involved in both the experimental and control groups. The overall odds ratio calculated was 3.63 [0.46, 28.87], but it failed to achieve statistical significance (*p* > 0.05) due to substantial variability (*I* ^2^ = 81%). The data suggest that BoNT‐A treatment may improve patient satisfaction; nonetheless, smaller studies may utilize different research approaches and present results in diverse ways. The odds ratio for all trials was 10.22 [2.39, 43.69]. Participants reported significant satisfaction following BoNT‐A injections (*Z* = 3.14, *p* = 0.002). Nonetheless, the significant variability (*I* ^2^ = 97%) highlights the necessity for careful scrutiny of the data [31]. Although most patients report satisfaction, the inconsistency in study results in a conclusion that is not particularly strong.

The forest plot highlights concerns regarding publication bias and discrepancies. The study data exhibits an uneven distribution, with a notable lack on the left side and a more substantial accumulation on the right, along with increased standard errors. Possible reasons for this may involve the influence of smaller studies or discrepancies in how results are reported. However, broader investigations at the higher end of the plot confirm the satisfaction rates among patients treated with BoNT‐A.

#### 3.3.2 Patient Satisfaction

The study concentrated on assessing patient satisfaction related to the administration of Botulinum Toxin A (BoNT‐A) in the upper facial region. The outcomes were positive; however, the studies showed considerable inconsistency. Subgroup 2.1.1 explored the distinctions between OnabotulinumtoxinA (the control) and the experimental treatments, IncobotulinumtoxinA or PrabotulinumtoxinA. The odds ratio obtained from three studies was 29.37 [1.17, 740.43]. As a result, individuals receiving OnabotulinumtoxinA exhibited diminished happiness levels in comparison to those treated with other formulations. However, the considerable variability (*I* ^2^ = 99%) and broad confidence interval led to reduced precision and heightened instability. The characteristics of patients, the techniques of injection, the dosages given, the definitions of “satisfaction,” and the advantages of cosmetics can vary significantly across different cultural settings [22].

In subgroup 2.1.2, two studies explored the effect of BoNT‐A on satisfaction; nonetheless, they failed to offer a comparative assessment. A total of 561 participants were involved in both the experimental and control groups. The overall odds ratio calculated was 3.63 [0.46, 28.87], but it failed to achieve statistical significance (*p* > 0.05) due to substantial variability (*I* ^2^ = 81%). The data suggest that BoNT‐A treatment may improve patient satisfaction; nonetheless, smaller studies may utilize different research approaches and present results in diverse ways.

The odds ratio for all trials was 10.22 [2.39, 43.69]. Participants reported significant satisfaction following BoNT‐A injections (*Z* = 3.14, *p* = 0.002). Nonetheless, the significant variability (*I* ^2^ = 97%) highlights the necessity for careful scrutiny of the data. Although most patients report satisfaction, the inconsistency in study results in a conclusion that is not particularly strong. This plot highlights concerns regarding publication bias and discrepancies [32]. The study data exhibit an uneven distribution, with a notable lack on the left side and a more substantial accumulation on the right, along with increased standard errors. Possible reasons for this may involve the influence of smaller studies or discrepancies in how results are reported. However, broader investigations at the higher end of the plot confirm the satisfaction rates among patients treated with BoNT‐A.

#### 3.3.3 Response Rate

The research focused on evaluating the response rates of different cosmetic Botulinum Toxin A (BoNT‐A) injection formulations to identify the most effective options for improving the aesthetics of the upper face. The study categorized the data into two distinct groups based on the specific type of BoNT‐A that was administered. In the initial group, five studies investigated Incobotulinumtoxin A, Prabotulinumtoxin A, and the well‐known Onabotulinumtoxin A. The experimental group consisted of 2277 participants, whereas the control group had 2262 individuals. The combined findings showed a Risk Ratio (RR) of 1.58 [95% CI: 1.09–2.29] with a *p*‐value of 0.02, suggesting statistical significance. This suggests that either Incobotulinumtoxin A or Prabotulinumtoxin A showed markedly better effectiveness in clinical environments when compared to Onabotulinumtoxin A [33]. Nonetheless, notable variability was detected (*I* ^2^ = 62%, *p* = 0.03), suggesting that the findings across the studies were inconsistent. The patients may exhibit a range of ages, the methods of injection administration could vary, the designs of the studies might be different, or the dosages of BoNT‐A used may not align consistently.

The second subgroup included three trials, with 713 participants in the experimental group and 716 individuals in the control group. The research explored various mixed or less familiar formulations of BoNT‐A, often known as BotN. The overall relative risk of 1.08 [95% CI: 0.89–1.32] for this subgroup demonstrated uniform results across all studies, exhibiting no variability (*I* ^2^ = 0%, *p* = 0.51) and not achieving statistical significance (*p* = 0.45). The control group comprised 2978 individuals, whereas the experimental group encompassed 2990 individuals. The eight trials showed low to moderate variability (*I* ^2^ = 63%), reached statistical significance (*p* = 0.02), and indicated an overall risk ratio of 1.40 [95% CI: 1.06–1.85]. The results suggest that unique or novel forms of BoNT‐A could improve the results of facial cosmetic treatments. It is important to confirm these results with further controlled and standardized experiments that include larger populations, given the noted inconsistencies [34].

#### 3.3.4 Overall Synthesis

This comprehensive examination evaluates findings from multiple clinical studies to assess the effectiveness of various types of Botulinum Toxin A (BoNT‐A) in treating cosmetic concerns in the upper facial region. The research examines three critical aspects: the severity of the wrinkles, the satisfaction levels of individuals, and the response rate among participants. The findings indicate that BoNT‐A demonstrates a predominantly positive impact across all metrics; however, the conclusions lack robustness and clarity due to the significant variability among the trials [35].

The administration of BoNT‐A injections appeared to reduce the visibility of wrinkles; however, the outcomes varied significantly (*I* ^2^ > 90%) due to inconsistencies in treatment efficacy, measurement methods, and the demographic characteristics of the sample population. In examining various groups, a notable increase in patient satisfaction with BoNT‐A was observed (pooled OR = 10.22, *p* = 0.002). The findings exhibited significant volatility (*I* ^2^ = 97%), a wide array of confidence levels, and indications of publication bias, suggesting that the aggregated estimates lacked reliability.

In comparison to OnabotulinumtoxinA, various newer or alternative BoNT‐A formulations, including IncobotulinumtoxinA and PrabotulinumtoxinA, demonstrated superior efficacy (RR = 1.58, *p* = 0.02). Considerable variation was observed (*I* ^2^ = 63%), primarily due to the differing methodologies and patient types employed across the studies. Certain studies utilizing novel formulations of BoNT‐A observed similar outcomes; however, the findings did not reach statistical significance [36].

BoNT‐A generally enhances the appearance of the upper face, particularly in terms of patients' happiness and responsiveness. However, the evidence lacks robustness due to its variability, the use of diverse methodologies, and potential biases in publication practices. It is essential to conduct additional high‐quality, standardized randomized controlled trials involving larger and more diverse populations to determine the efficacy of BoNT‐A in comparison to other therapies and to assess the consistency of its aesthetic outcomes across various clinical contexts.

## 4 Discussion

This study indicates that the use of botulinum neurotoxin type A (BoNT‐A) in the upper facial region effectively reduces wrinkles and enhances patient satisfaction. The application of BoNT‐A notably enhanced the effectiveness of wrinkle elimination and increased overall satisfaction with the treatment when contrasted with the absence of any intervention. In randomized trials, 97% of patients receiving BoNT‐A experienced a one‐grade improvement in glabellar lines within 1 month, whereas only 20% of those in the placebo group saw similar results. The findings indicate a notable impact of BoNT‐A, with odds ratios of 21 for enhancement in wrinkles and 10 for satisfaction (*p* < 0.01), thereby confirming the results of the study [37]. A Cochrane trial demonstrated that BoNT‐A was more effective than placebo in reducing glabellar lines after a 4‐week period. Chang et al. observed that the treatment led to a 28% increase in patient satisfaction, which corresponded with the improvements in wrinkles. To sum up, our results are consistent with earlier studies: BoNT‐A significantly diminishes the appearance of wrinkles and enhances overall satisfaction [38, 39].

Our results are consistent with earlier studies. The cutaneous effects of onabotulinumtoxinA, incobotulinumtoxinA, and abobotulinumtoxin A are comparable. Michaels et al. found that both Dysport and Botox effectively diminished upper face wrinkles in a comparable manner. In comprehensive research, Researcher found that incobotulinumtoxinA and onabotulinumtoxinA were equally effective in treating glabellar lines [40]. Our subgroup analysis did not reveal a definitive leader. Both subgroups of incobotulinumtoxinA/prabotulinumtoxinA and onabotulinumtoxinA, as well as the BoNT‐A versus control group, showed positive outcomes. Recent Phase III trials indicate that the efficacy of newer BoNT‐A drugs is comparable to that of their older counterparts. Higgins et al. [28] demonstrated that Botulax is both effective and safe, comparable to onabotulinumtoxinA. The response rates observed were quite comparable, standing at 88.5% and 87.4% respectively. Chang et al. found that treatments with BoNT‐A that reduced wrinkles led to higher patient satisfaction. This comprehensive examination enhances both clinical and systematic evaluations: the injections of BoNT‐A reliably diminish wrinkles in the upper face, resulting in satisfied clients [31].

Examining the influence of publication bias and the importance of diversity The findings exhibited considerable variability (*I* ^2^ > 90%). Elevated *I* ^2^ values suggest that variations in trial outcomes lead to changes in effect sizes rather than being attributed to random chance. The trials involving BoNT‐A exhibit variability due to factors such as the types of toxins used, the dosages administered, the protocols for injection, the measures of outcomes, the demographics of the patients, and the durations of follow‐up. Higgins et al. indicate that factors such as dosage, injection placement, patient weight, and wrinkle severity influence the outcomes. Kroumpouzos et al. point out that the significant variability among the articles on this subject is due to differences in authorship, management, and treatment approaches [41]. The overall findings suggest that BoNT‐A offers advantages, yet the intricacies of real‐world scenarios influence the interpretations of the studies. Random‐effects models were utilized to address variability. Comparisons featuring substantial odds ratios and confidence intervals exhibit broad ranges. The findings exhibited a lack of precision attributed to the variances present [42].

Our results may be influenced by potential publication biases. Funnel plots revealed the presence of asymmetry. This could suggest that studies with minimal impact or solely favorable outcomes are being highlighted. According to Gold's test benchmarks, funnel asymmetry is observed in 38% of traditional analyses. It is important to exercise caution, as both clinical and random factors can lead to asymmetrical funnels rather than solely attributing it to publishing bias. Kroumpouzos suggests that cosmetic remedies have their value [43]. This suggests that research could yield favorable results, potentially exaggerating effectiveness assessments. Given these challenges, it is essential to place data in context. BoNT‐A demonstrates effectiveness; nonetheless, the exclusion of less favorable studies could potentially inflate its perceived advantages.

The organization of trials and the variation in stated outcomes created challenges for making comparisons. The research employed different measurement scales (4‐point versus 5‐point wrinkle scales), classified responders in various ways (≥ 1‐grade versus ≥ 2‐grade improvement), and evaluated outcomes over differing time frames (4–6 weeks, sometimes extending beyond 3 months). The Cochrane review pointed out that the trials employed different toxins, dosages, treatment cycles, and facial areas, which complicates the synthesis of results. Our results were consistent with earlier studies [32]: Various studies utilized Merz Aesthetics, while others focused on the Facial Wrinkle Scale. Patients assessed their satisfaction through the FLTSQ and FACE‐Q, yet the results varied. Respondents had varying definitions of what constitutes a “response.” Certain studies categorized responses as “none/mild” wrinkles, while others employed relative improvements. Altering processes can create an impression of change, even when the underlying reality remains unchanged, leading to confusion in interpreting outcomes [44].

This indicates that physicians perceive the content as beneficial, even in light of cautions. The findings indicate that BoNT‐A injections reliably diminish wrinkles in the upper face. Healthcare professionals might have confidence in BoNT‐A to bring about significant changes in patients. About 90% of individuals with glabellar frown lines experience an improvement of one grade each month. Positive outcome: patients express satisfaction [26]. A significant number of studies indicate that more than 90% of patients express satisfaction following therapy. Individuals express a sense of rejuvenation and authenticity, enhancing their self‐assurance. Regardless of the BoNT‐A product used, Chang et al. observed an increase in satisfaction [45]. This indicates that choices regarding Botox, Dysport, Xeomin, and other BoNT‐As may be influenced by both cost and effectiveness, rather than effectiveness alone. Michaels et al. suggest that AbbotulinumtoxinA (Dysport) effectively reduces wrinkles similarly to onabotulinumtoxinA, but at a lower cost. Kroumpouzos et al. investigate the processes related to immunogenicity. First‐generation toxins such as Botox and Dysport may be neutralized by antibodies. Xeomin and Daxi‐BoNT are devoid of proteins and could potentially lower this risk [46]. In cases where the initial treatment does not yield the desired results, incobotulinumtoxinA could be considered as an alternative option. Factors related to long‐term care are significant. According to the study, most BoNT‐As demonstrate a notable effect lasting between 4 and 6 months. Counseling ought to encompass re‐treatments every 3–4 months, along with a gradual approach to wrinkle elimination.

What are the next steps? Our findings indicate that numerous areas require additional investigation. It is essential to conduct additional high‐quality randomized controlled trials that compare different formulations of BoNT‐A, particularly in areas beyond the glabellar lines. The Cochrane reviewers emphasize the importance of assessing long‐term outcomes and making comparisons among the most prevalent types of BoNT‐A in upcoming trials. Saybel et al. conducted research on crow's feet, while Xie carried out a study in China. Research on forehead lines and various demographics is quite limited. Comparative effectiveness studies using network meta‐analyses could encompass all five BoNT‐A medications. Standardizing measurements would enhance reliability as well. It is essential to reach a consensus on the thresholds for responders (is a 1‐point improvement sufficient?) and to employ validated patient‐reported measures such as the FACE‐Q to evaluate happiness and quality of life. The significance of long‐term safety and antibody production cannot be overstated. It is important to investigate ptosis, asymmetry, and the role of antibody‐mediated resistance, particularly in the context of multiple injections. Ultimately, research needs to pinpoint the emotional and behavioral factors that can serve as indicators [47]. The outcomes could vary based on factors such as age, skin type, and muscle mass. Tailoring treatment approaches could be enhanced through the refinement of subgroups or injection techniques.

The data reinforces the findings from clinical research: beauty products. Patients appreciate upper‐face BoNT‐A injections for their effectiveness in reducing wrinkles. Thorough examination of the data is essential due to the significant variability among the studies [48]. A robust methodology and comparative studies will enhance the evidence of discrepancies. This information can assist healthcare professionals in tailoring BoNT‐A and treatment approaches. It is essential to comprehend the primary advantages of facial rejuvenation.

### 4.1 Limitations

The new section includes the following key points:

- Heterogeneity among included studies, especially regarding injection techniques, doses, and outcome measures, which may influence pooled estimates.
- Limited number of high‐quality randomized controlled trials, with many studies being observational or small in sample size.
- Variability in outcome reporting, including differences in patient satisfaction scoring systems and response definitions across trials.
- Short follow‐up periods in most studies limit long‐term assessment of efficacy and safety.
- Potential publication bias, as aesthetic studies with negative or nonsignificant outcomes may be underreported.

These limitations are now clearly articulated to ensure transparency and assist readers in interpreting the findings appropriately.

## Funding

The authors have nothing to report.

## Disclosure

Permission to reproduce material from other sources: While no permission for reproduction was required, we remain committed to accurate citation and appropriate attribution of all sourced materials to maintain the highest standards of academic integrity and scholarly discourse.

## Ethics Statement

This systematic review and meta‐analysis was conducted in accordance with PRISMA 2020 guidelines. All analyses were based on previously published studies; therefore, no new ethical approval or patient consent was required.

## Consent

The authors have nothing to report.

## Conflicts of Interest

The authors declare no conflicts of interest.

## Supporting information


**Appendix S1:** jocd70655‐sup‐0001‐AppendixS1.docx.

## Data Availability

The data that support the findings of this study are available from the corresponding author, Dr. Alaa Safia, upon reasonable request.
