# Supplementary material for: Cosmetic Botulinum Toxin A Injections to the Upper Face: A Systematic Review and Meta‐Analysis of Clinical Studies
Source: J Cosmet Dermatol. 2026 Jan 8;25(1):e70655. doi: 10.1111/jocd.70655 (PMC12783872; doi:10.1111/jocd.70655)
Supplement: Supplementary file 1 — Appendix S1: jocd70655‐sup‐0001‐AppendixS1.docx. [file JOCD-25-e70655-s001.docx]

# Supplementary Material

## Appendix S1. Search Strategy, Study Selection, and Methodological Assessment

### Electronic Search Strategy

A comprehensive literature search was performed to identify all relevant clinical studies evaluating cosmetic botulinum toxin type A injections to the upper face. The following electronic databases were searched:

• PubMed/MEDLINE
• Embase
• Web of Science
• Cochrane Central Register of Controlled Trials (CENTRAL)

The search covered the period from database inception to the final search date. Both Medical Subject Headings (MeSH) and free-text terms were used. The search strategy included terms related to botulinum toxin type A, cosmetic or aesthetic indications, and upper facial regions. Search strategies were adapted for each database, and reference lists of included studies were manually screened.

### Study Selection

Study selection was conducted in accordance with the PRISMA 2020 guidelines. After removal of duplicate records, titles and abstracts were screened independently by two reviewers. Full-text articles were assessed for eligibility. Disagreements were resolved through discussion or consultation with a third reviewer.

### Eligibility Criteria

Inclusion criteria included clinical studies evaluating cosmetic botulinum toxin type A injections to the upper face in adult participants. Exclusion criteria included case reports, small case series, reviews, editorials, animal studies, non-cosmetic indications, and studies without extractable data.

### Data Extraction

Data were extracted independently by two reviewers using a standardized extraction form. Extracted data included study characteristics, participant demographics, type and dose of botulinum toxin A, injection technique, follow-up duration, outcomes, and adverse events.

### Risk of Bias Assessment

Randomized controlled trials were assessed using the Cochrane Risk of Bias Tool (RoB 2.0), while non-randomized studies were assessed using the ROBINS-I tool. Any disagreements were resolved by consensus.

### Statistical Analysis

Meta-analysis was conducted using a random-effects model. Effect estimates were reported as risk ratios or mean differences with 95% confidence intervals. Heterogeneity was assessed using the I² statistic, and publication bias was evaluated when appropriate.

### Appendix S1 Caption

Appendix S1. Detailed description of the electronic search strategy, study selection process, eligibility criteria, data extraction methods, risk of bias assessment, and statistical analysis applied in the systematic review and meta-analysis of cosmetic botulinum toxin type A injections to the upper face.
